# Characteristics and risk factors of mortality in patients with systemic sclerosis-associated interstitial lung disease

**DOI:** 10.1080/07853890.2023.2179659

**Published:** 2023-02-22

**Authors:** Hye Jin Jang, Ala Woo, Song Yee Kim, Seung Hyun Yong, Youngmok Park, Kyungsoo Chung, Su Hwan Lee, Ah Young Leem, Sang Hoon Lee, Eun Young Kim, Ji Ye Jung, Young Ae Kang, Young Sam Kim, Moo Suk Park

**Affiliations:** a Division of Pulmonary and Critical Care Medicine, Department of Internal Medicine, Myongji Hospital, Hanyang Medical Center, Gyeonggi-do, Republic of Korea; b Division of Pulmonary and Critical Care Medicine, Department of Internal Medicine, Severance Hospital, Yonsei University College of Medicine, Seoul, Republic of Korea

**Keywords:** Systemic sclerosis, interstitial lung disease, autoimmune disease, fibroblast

## Abstract

**Background:**

Systemic sclerosis (SSc) is a heterogeneous autoimmune disease characterized by dysregulation of fibroblast function, which often involves the lungs. Interstitial lung disease (ILD) associated with SSc (SSc-ILD) is a major cause of death among patients with SSc. Our study aimed to identify risk factors for mortality and compare the clinical characteristics of patients with SSc-ILD.

**Patients and methods:**

Patients were retrospectively enrolled between 2010 and 2018 in a tertiary hospital in Korea. Patients with SSc-ILD were classified depending on the first pulmonary function test or radiologic findings: extensive (*n* = 46, >20% disease extent on computed tomography (CT) or forced vital capacity [FVC] < 70% in indeterminate cases) and limited (*n* = 60, <20% disease extent on CT or FVC ≥70% in indeterminate cases).

**Results:**

Patients in the extensive group were younger (mean age ± SD 49.3 ± 11.5) than those in the limited group (53.9 ± 12.5, *p* = .067) at diagnosis. The extensive group showed frequent pulmonary hypertension (43.5% vs. 16.7%, *p* = .009) and higher erythrocyte sedimentation rate (61.3 ± 33.7 vs. 42.1 ± 26.0, *p* = .003) and mortality (32.6%, mean duration of follow-up, 100.0 ± 44.7 months vs. 10.0%, 86.0 ± 53.4 months, *p* = .011). ILD was detected within five years from the first visit (median years 3.5 (1.0, 6.0) vs. 4.5 (0.6, 9.0), survivors vs. non-survivors), and mortality occurred in 19.8% of all patients during a 15-year follow-up. Older age, lower FVC, and initial disease stage (limited or extensive) were associated with mortality, but FVC decline was similar in the limited and extensive groups, such as 15–20% in the first year and 8–10% in the next year, regardless of the initial extent of the disease.

**Conclusions:**

Approximately 10% of patients with SSc-ILD in the limited and extensive group showed progression. ILD was detected at a median of less than five years from the first visit; therefore, it is necessary to carefully monitor patients’ symptoms and signs from an early stage. Long-term surveillance is also required.

**Key messages**
- Patients with systemic sclerosis-interstitial lung disease manifested a heterogeneous disease course.
- Approximately 10% of the patients in the limited group showed progression, which was similar to the proportion of patients in the extensive group.
- Interstitial lung disease was detected at a median of less than five years from the first visit.

## Introduction

As a heterogeneous autoimmune disease that is characterized by dysregulation of fibroblast function, systemic sclerosis (SSc) causes multi-organ damage, often involving the lungs [1,2]. Interstitial lung disease (ILD) associated with SSc (SSc-ILD) is a major cause of death in patients with SSc. Typically, 50% of patients with SSc progress to develop clinically significant ILD, usually within the first five years after the SSc diagnosis [3]. SSc-ILD results from the interplay between fibrosis, autoimmunity, inflammation, and vascular injury [1]. Activation of the immune system promotes fibroblast recruitment and activation, extracellular matrix overproduction, and replacement of normal pulmonary architecture by scarring [4,5]. The incidence of complications is associated with disease severity and progression, with approximately 25% of patients showing severe lung involvement within 3 years of an SSc diagnosis [6]. The prevalence of SSc-ILD has been found to range from 26% to 86%, depending on the region, study quality, classification criteria, and publication year [7]. ILD is a major contributing factor to the morbidity and mortality associated with SSc [2]. Patients who are male, current smokers, and of an older age at presentation have an increased risk of disease progression and early mortality [8–10]. However, the clinical course is variable, and there is insufficient information about the disease, especially in Asians. Our study aimed to identify the risk factors for mortality and describe the clinical characteristics of patients with SSc-ILD in Korea.

## Methods

### Ethics statement

The study protocol was approved by the institutional review board of Severance Hospital, South Korea (IRB No. 4-2021-0227). The study design was approved by the appropriate ethics review board. None of the patient data had identifying information, and the requirement to obtain informed patient consent was waived by the IRB.

### Patient recruitment

**Figure 1.**
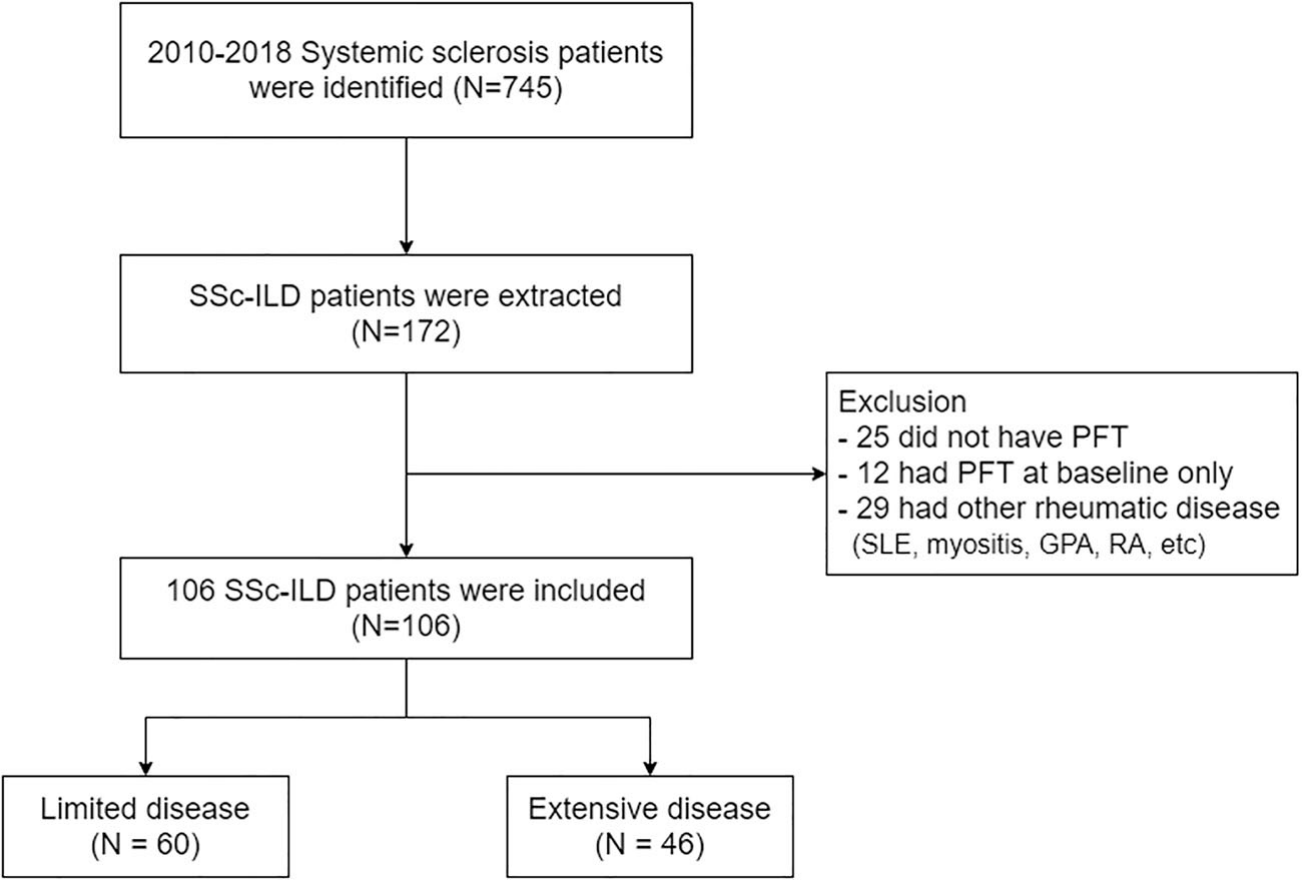
Flowchart of patient recruitment.

Figure 1 presents a flow chart of the patient recruitment process. Initially, data for 745 patients between 2010 and 2018 were extracted using the International Classification of Diseases 10^th^ Revision (ICD-10) codes M34.0 (progressive SSc), M34.1 (CRIST syndrome), M34.2 (SSc induced by drugs and chemicals), M34.8 (other forms of SSc), and M34.9 (SSc, unspecified). We identified 172 patients with SSc who met the American College of Rheumatology/European Alliance of Associations for Rheumatology 2013 criteria with ILD through a computed tomography (CT) scan. We excluded 37 patients who did not undergo at least two pulmonary function tests (PFTs) after ILD diagnosis to evaluate disease progression and 29 patients with other rheumatic diseases. The 106 patients included in this study were divided into two groups: limited and extensive disease groups. Regarding the difference between SSc and ILD diagnosis time, baseline information was collected based on ILD diagnosis.

### Pulmonary function test and high-resolution computed tomography (HRCT)

We defined the severity of SSc-ILD based on the ‘Goh criteria’ [11]. The limited disease was defined as minimal disease as determined by high-resolution CT (HRCT) (<20% disease extent or a forced vital capacity [FVC] of ≥70% in indeterminate cases). The extensive disease was defined as a severe disease on HRCT (>20% disease extent with an FVC of <70% in indeterminate cases). Progression was defined as an absolute FVC decline of >10% from the baseline (the time of ILD diagnosis) or a 5–9% FVC decline in association with a diffusing capacity for carbon monoxide (DLco) decline of more than 15%.

### Echocardiogram

Echocardiograms were used to screen for pulmonary hypertension (PH) or cardiac involvement. PH is defined as a mean pulmonary arterial pressure ≥25 mmHg, as confirmed by right heart catheterization [12]. Due to its invasiveness and feasibility in clinical settings, traditional echocardiogram-estimated right ventricular systolic pressure (RVSP) values >40 mmHg were used to categorize suspected PH [12,13].

### Serologic markers

Serologic markers, including anti-centromere, anti-RNA polymerase, and anti-topoisomerase I (anti-Scl-70) antibodies (Ab) and the erythrocyte sedimentation rate (ESR), were checked for in the initial evaluation. Systemic symptoms such as dyspnea, dysphagia, joint pain, or skin ulcers were reviewed in the written medical records.

### GAP score

The GAP model predicts mortality risk in chronic ILD [14]. This risk was calculated for all patients based on sex (0–1 point), age (0–2 points), FVC (0–2 points), and DL_CO_ (0–3 points). The disease was classified as stage I (0–3 points), II (4–5 points), or III (6–8 points).

### Computed tomography (CT) assessment

The quality of CT images was assessed by an observer with at least 10 years of experience in chest CT imaging who was blinded to the clinical findings. The CT extent was graded by three radiologists, one trainee, and two experts with more than 24 months of experience in interstitial lung disease. The radiologists independently reviewed CT images, and any inconsistencies were resolved through discussion and agreement.

### Statistical analysis

Categorical variables were numerically described as percentages. These were compared using the chi-squared test; continuous variables were compared using either an independent *t*-test or Mann–Whitney U test, according to the normality of distribution. Continuous variables with normal distribution were reported as means with standard deviations or medians with interquartile ranges. Declines in FVC and DLco values were calculated using the results of PFTs at the time of SSc diagnosis and 1 year after the initial SSc diagnosis. Survival probability was estimated using the Kaplan–Meier method for patients with and without progressive disease. Statistical significance was determined using a log-rank test. Risk factors for mortality were identified through logistic regression analysis, with careful selection of variables with a *p*-value of <.1 and demographic factors selected after univariate regression analysis. All statistical analyses were performed using IBM SPSS Statistics (version 25; IBM Corp., Armonk, NY, USA).

## Results

### Baseline characteristics of patients

We screened 745 patients using ICD-10 codes, of which 172 patients with SSc-ILD were enrolled. The overall prevalence of SSc-ILD among SSc patients was 23.1%, according to the patient recruitment chart. Twenty-nine patients, constituting approximately 20% of the total population, were excluded because they had other rheumatic diseases. The baseline characteristics of the 106 patients with SSc-ILD, who were finally included in the study, are presented in Table 1. There was a higher proportion of female sex in both groups. Patients in the extensive group were younger at diagnosis (mean age ± SD, 49.3 ± 11.5) than those in the limited group (53.9 ± 12.5, *p* = .067). No significant differences in the presence of autoimmune markers, such as anti-centromere Ab, anti-RNA polymerase Ab, and anti-Scl-70 Ab, were found between the two groups. The median time of ILD development from the first visit was 3.0 and 4.0 years in the limited and extensive groups, respectively. The extensive group showed frequent pulmonary hypertension at 43.5% compared to 16.7% in the limited group. The extensive group also had significantly higher ESR levels (61.3 ± 33.7 vs. 42.1 ± 26.0, *p* = .003) and higher mortality (32.6% vs. 10.0%, *p* = .011) compared to the limited group. The most common causes of death were pneumonia (*n* = 6) and malignancy (*n* = 7; two cases of mesothelioma and five cases of lung cancer), followed by ILD-acute exacerbation (ILD-AE) (*n* = 2), pulmonary artery hypertension (*n* = 1), myocarditis (*n* = 1), sepsis (*n* = 1), renal crisis (*n* = 1), post-procedural event (*n* = 1), and sudden cardiac death (*n* = 1).

**Table 1.** Baseline characteristics of patients.

Variable	Limited	Extensive	Total	*p*-value
	(*n* = 60)	(*n* = 46)	(*n* = 106)	
Age, years	53.9 ± 12.5	49.3 ± 11.5	51.9 ± 12.7	.067
Sex, men	7 (11.7)	7 (15.2)	14 (13.2)	.773
Smoking exposure, *n* (%)				.813
Never	39 (88.6)	38 (88.4)	77 (72.6)	
Former	1 (2.3)	3 (7.0)	4 (3.8)	
Current	4 (9.1)	2 (4.7)	6 (5.7)	
Pack-years	2.6 ± 10.0	2.8 ± 8.1	2.7 ± 9.1	.831
FVC % predicted	86 ± 14	54 ± 14	71.9 ± 21.3	<.001
FEV_1_ % predicted	96 ± 17	62 ± 16	81.3 ± 23.9	<.001
DL_CO_, % predicted	75 ± 20	54 ± 22	65.3 ± 23.6	<.001
Progression within 1st year	12 (20.0)	7 (15.2)	19 (17.9)	.726
Anti-centromere Ab, *n* (%)	4 (9.5)	2 (6.3)	6 (5.7)	.693
Anti-RNA polymerase, *n* (%)	10 (23.8)	8 (24.2)	18 (17.0)	.999
Anti-Scl 70, *n* (%)	35 (66.0)	25 (56.8)	60 (56.6)	.404
Reflux/dysphagia symptoms, *n* (%)	23 (38.3)	23 (50.0)	46 (43.4)	.243
Digital ulcers, *n* (%)	46 (76.7)	40 (87.0)	86 (81.1)	.216
Synovitis, joint symptoms, *n* (%)	29 (48.3)	20 (43.5)	49 (46.2)	.843
ILD related respiratory symptoms	42 (70.0)	39 (84.8)	81 (76.4)	.585
ESR (mm/h)	42.1 ± 26.0	61.3 ± 33.7	50.5 ± 31.0	.003
Pulmonary hypertension*	10 (16.7)	20 (43.5)	30 (28.3)	.009
Immunosuppressant use, *n* (%)				
Steroid	44 (73.3)	37 (80.4)	81 (76.4)	
Azathioprine	12 (20.0)	13 (28.3)	25 (23.6)	
Cyclophosphamide	1 (1.7)	7 (15.2)	8 (7.5)	
Mycophenolate mofetil	2 (3.3)	3 (7.0)	5 (4.7)	
Rituximab	0 (0.0)	0 (0.0)	0 (0.0)	
Methotrexate	5 (8.3)	2 (4.3)	7 (6.6)	
Antifibrotic drugs				
D-penicillamine	25 (41.7)	14 (30.4)	39 (36.8)	
Underlying malignancy^†^	14 (21.7)	8 (24.2)	21 (19.8)	.481
Follow-up period (month)	100.0 ± 44.7	86.0 ± 53.4	92.8 ± 49.3	N/A
Time to ILD development from the first visit (year)	3.0 (1.0, 6.0)	4.0 (1.5, 7.0)	4.0 (1.0, 7.0)	.377
Mortality	6 (10.0)	15 (32.6)	21 (19.8)	.011

ILD: interstitial lung disease; ESR: erythrocyte sedimentation rate; FVC: forced vital capacity; FEV1: forced expiratory volume in 1 s; DLco: diffusing capacity of carbon monoxide. *Pulmonary hypertension was defined using traditional echocardiogram-estimated right ventricular systolic pressure (RVSP) >40 mmHg.

^†^Malignancy: (1) limited group: lung cancer (*n* = 5), thyroid cancer (*n* = 3), breast cancer (*n* = 4), cervical cancer (*n* = 2); (2) extensive group: lung cancer (*n* = 3), thyroid cancer (*n* = 1), breast cancer (*n* = 1), ovarian cancer (*n* = 1), colon cancer (*n* = 1), ampulla of Vater cancer (*n* = 1).

Most patients were treated with immunosuppressive drugs such as steroids (76.4%), azathioprine (23.6%), cyclophosphamide (7.5%), mycophenolate mofetil (MMF) (7.5%), and methotrexate (MTX) (6.6%). D-penicillamine was used as adjunctive therapy (36.8%).

Table 2 details the clinical characteristics of survivors and non-survivors. Those who did not survive were older than those who survived (mean age 56.9 ± 13.4 vs. 50.7 ± 12.3, *p* = .049, non-survivors vs. survivors) and had a higher proportion of male sex (30.0% vs. 9.3%, *p* = .024, non-survivors vs. survivors). FVC, forced expiratory volume in one second, and DLco were significantly lower in the non-survivor group than those in the survivor group; however, autoimmune antibodies and systemic symptoms were not statistically different between the groups. RVSP was significantly higher in the non-survivor group than that in the survivor group (32.3 ± 16.8 vs. 44.7 ± 25.3, *p* = .005). The proportion of malignancy was higher in the non-survivor group than that in the survivor group (33.3% vs. 16.5%, *p* = .182). The median time to ILD development was 3.5 (1.0, 6.0) years and 4.5 (0.6, 9.0) years in the survival and non-survival groups, respectively.

**Table 2.** Clinical characteristics between survivors and non-survivors in patients with SSc-ILD.

Variable	Survivors (*n* = 85)	Non-survivors (*n* = 21)	*p*-value
Age, years	50.7 ± 12.3	56.9 ± 13.4	.049
Sex, men	8 (9.4)	6 (28.6)	.031
Smoking exposure, *n* (%)			.449
Never	60 (70.6)	18 (85.7)	
Former	3 (3.5)	2 (9.5)	
Current	6 (7.1)	1 (4.8)	
Pack-years	18.7 ± 32.3	2.0 ± 6.3	.786
FVC % predicted	74.4 ± 20.7	61.8 ± 21.1	.010
FEV_1_ % predicted	83.6 ± 23.6	72.3 ± 23.5	.033
DL_CO_, % predicted	69.8 ± 22.2	47.3 ± 20.5	<.001
GAP score	0.6 ± 0.9	1.3 ± 1.4	.056
Anti-centromere Ab, *n* (%)	3 (3.5)	3 (14.3)	.111
Anti-RNA polymerase, *n* (%)	16 (18.8)	2 (9.5)	.337
Anti-Scl 70, *n* (%)	49 (57.6)	11 (52.4)	.606
Reflux/dysphagia symptoms, *n* (%)	36 (42.4)	10 (47.6)	.806
Digital ulcers, *n* (%)	72 (84.7)	14 (66.7)	.069
ILD related respiratory symptoms	63 (74.1)	18 (85.7)	.391
ESR (mm/h)	48.0 ± 30.4	61.6 ± 31.8	.090
RVSP (mmHg)	32.3 ± 16.8	44.7 ± 25.3	.005
Underlying malignancy^†^	14 (16.5)	7 (33.3)	.182
Extensive group	29 (34.1)	15 (71.4)	.002
Follow-up period (month)	96.4 ± 47.0	80.4 ± 56.3	N/A
Time to ILD development from first visit (year)	3.5 (1.0, 6.0)	4.5 (0.6, 9.0)	.361

ILD: interstitial lung disease; ESR: erythrocyte sedimentation rate; FVC: forced vital capacity; FEV1: forced expiratory volume in 1 s; DLco: diffusing capacity of carbon monoxide; RVSP: right ventricular systolic pressure; GAP score, sex (G), age (A), physiology (P).

^†^Malignancy: (1) Survivors: lung cancer (*n* = 3), thyroid cancer (*n* = 3), breast cancer (*n* = 5), cervical cancer (*n* = 2), colon cancer (*n* = 1); (2) Non-survivors: lung cancer (*n* = 5), thyroid cancer (*n* = 1), ovarian cancer (*n* = 1), ampulla of Vater cancer (*n* = 1).

Table 3 shows the results of the logistic regression analysis to determine risk factors for mortality. In the univariate analysis, the factors related to death were age (odds ratio [OR] 1.043, *p* = .043), male sex (OR 4.179, *p* = .020), baseline FVC (OR 0.970, *p* = .017), limited or extensive stage (OR 5.483, *p* = .003), and high RVSP (OR 1.028, *p* = .043). Baseline FVC was used as a variable in the multivariate analysis shown in model 1, and the initial limited or extensive stage was included as a variable in model 2, separately. The multivariate analysis determined that advanced age, initial limited or extensive stage and low FVC% (predicted) were significantly associated with mortality.

**Table 3.** Multivariable logistic regression models for the risk of mortality in patients with SSc-ILD.

	Univariate		Multivariate model 1		Multivariate model 2	
Variables	OR (95% CI)	*p*-value	OR (95% CI)	*p*-value	OR (95% CI)	*p*-value
Age, years	1.043 (1.001–1.086)	.043	1.063 (1.015–1.113)	.009	1.058 (1.009–1.110)	.020
Sex, men	4.179 (1.256–13.897)	.020	3.311 (0.932–11.765)	.064	3.138 (0.814–12.096)	.097
FVC % predicted	0.970 (0.946–0.995)	.017	0.958 (0.931–0.986)	.004		
Limited/extensive stage	5.483 (1.809–16.616)	.003			7.802 (2.236–27.228)	.001
Anti-centromere Ab	4.231 (0.765–23.407)	.098				
Anti-Scl 70 Ab	0.622 (0.226–1.713)	.358				
ESR	1.016 (0.999–1.032)	.062				
RVSP (mmHg)	1.028 (1.001–1.056)	.043				
Underlying malignancy	2.549 (0.870–7.464)	.088				

FVC: forced vital capacity; ESR: erythrocyte sedimentation rate; RVSP: right ventricular systolic pressure.

Table 4 shows the disease course according to the initial disease stage. Approximately 20% of patients in the limited group and 15.2% in the extensive group showed a progressive disease course in the first year of follow-up. FVC declines were similar in the limited and extensive groups, at 15–20% in the first year; however, approximately 8–10% of patients showed progressive lung function decline regardless of the initial disease extent.

**Table 4.** Disease course according to initial disease stage.

Variable	First 12-month period with FVC decline		After 2nd year period with FVC decline	
Overall FVC decline	Progression	Stable	Progression	Stable
Extensive (*n* = 46)	7 (15.2)	39 (84.8)	5 (10.7)	41 (89.1)
Limited (*n* = 60)	12 (20.0)	48 (80.0)	5 (8.3)	55 (91.7)

FVC: forced vital capacity.

**Figure 2.**
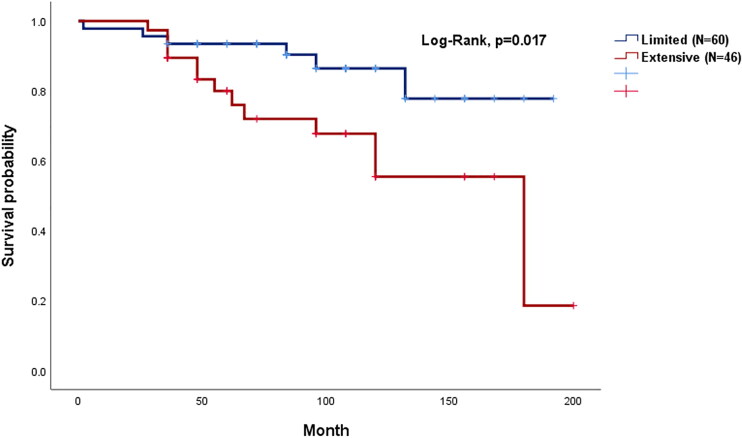
Kaplan–Meier curves of all-cause mortality between limited and extensive groups.

Kaplan–Meier survival curves (Figure 2) estimated all-cause mortality in the limited and extensive groups. The extensive group showed significantly higher mortality during the follow-up period (log-rank test, *p* = .017).

## Discussion

This study revealed the disease course, clinical characteristics, and prognosis of patients with SSc-ILD in South Korea. The data, which described an SSc-ILD prevalence rate of 23.1%, were collected from a tertiary hospital with a mean follow-up period of 8 years. Moreover, similar declines in FVC were observed in the limited and extensive groups, at 15–20% in the first year and 10% in the second year after diagnosis; however, this may not reflect all SSc-ILD data in Korea.

SSc is a complex and heterogeneous autoimmune disease; therefore, epidemiologic studies are important. However, its presentation varies depending on ethnicities and regions. African-Americans are more likely to develop SSc than Caucasians and experience worse pulmonary disease and greater morbidity [15]. A similar phenomenon was observed in East Asia, where a significantly higher prevalence of SSc-ILD was reported in China than in Japan and Korea [16].

The most common SSc-related comorbidity is ILD, which is present in 29.3% of patients with SSc in Japan with a mean age of 40–60 years at the time of diagnosis and a female predominance [17]. Approximately 25% of patients developed significant pulmonary involvement within 3 years of SSc diagnosis in the USA [6], and up to 35% of patients with SSc developed SSc-ILD in Europe [18]; these results are consistent with our data. Another study showed that parenchymal lung involvement often appears early after an SSc diagnosis, with 25% of patients developing clinically significant ILD within 3 years, as defined by physiological or radiographic findings [6]. This study also showed similar results, with an SSc-ILD prevalence of 23.1% and its development at a mean of 3.7 years after the SSc diagnosis.

Pulmonary involvement, known as SSc-ILD, is the major cause of death in SSc; therefore, evaluating and intervening in ILD in the early stages of SSc is important. The variable clinical course of SSc-ILD suggests that patients should be well monitored for symptoms and lung function decline. However, there is no consensus on the choice of diagnostic tools or the frequency of monitoring [19]. Continuous measurement of symptoms such as the appearance or worsening of dyspnea, dysphagia, or cough; physical examinations for sclerodactyly, Raynaud phenomena, or digital ulcers; and PFTs are important assessment tools for monitoring disease progression at regular clinic visits [4,20]. During regular visits, various clinical features were observed without a uniform pattern of worsening and improvement in lung function. There have been studies on the risk factors for the presence of ILD in patients with SSc, such as anti-Scl-70 Ab, ESR, and the development of PAH [21]; however, data on patients with SSc-ILD are limited. Currently, biomarkers such as interleukin-6 [22], Krebs von den Lungen (KL)-6 [23], and CCL-2 [24] are known to be associated with the progression of SSc-ILD. KL-6 is predominantly expressed in the lungs and is associated with lung damage, fibrosis, and inflammation [25,26]. However, it is difficult to apply these novel biomarkers regularly in the clinical setting because of their cost and unavailability.

Our study showed that advanced age, initial disease stage (limited or extensive), and underlying malignancies were significantly associated with mortality in the multivariate analysis. Assessment of the outcomes of patients with SSc-ILD is limited because of the rarity of the disease and the lack of long-term data. Moreover, there is a lack of consensus regarding the best outcome measures for assessing the course and progression of ILD, as with other connective tissue disease-related ILDs [27]. Our study found a long-term clinical difference in disease progression or mortality of SSc-ILD according to the initial disease stage.

Many studies have investigated risk factors for mortality in retrospective and prospective SSc cohorts [11,28,29]. In the present study, older age [30], male sex [31], lower FVC [11], and a high level of RVSP [32] were consistent risk factors in the univariate logistic regression model. According to the disease outcomes, surveillance of pulmonary hypertension and malignancy is as important as lung function decline; therefore, CT scans and PFTs should be performed regularly in patients with SSc-ILD in outpatient clinics.

A previous study showed anti-centromere Ab to be associated with better survival (hazard ratio [HR] 0.62; 95% CI, 0.47–0.82; *p* = .025) and anti-Scl-70 Ab with worse survival (HR, 1.38; 95% CI, 1.09–1.74; *p* = .022) [33]. Anti-Scl-70 Ab is found in 20–30% of patients with SSc in many ethnic groups; however, this proportion is higher in Europe (40–60%) [34]. It is associated with a high (60%) risk of ILD, regardless of the extent of skin thickening (diffuse or limited) [35]. In our study, 56.6% of the patients with SSc-ILD had this antibody; however, it did not show clinical significance in terms of mortality.

During the first follow-up year, the limited group showed a higher proportion of progression (20%) than the extensive group (15.2%). This is probably because the first medical intervention may have been delayed. In the second follow-up year, the extensive group showed a higher proportion; however, most patients had a stable disease course. In the late phase of fibrosis, it was linked with poor outcomes in patients with SSc-ILD due to other complications, such as PH [36]. This could result from altered lung structure, suggesting a generally less rapid but similar progression pattern to IPF [37]. This is the most appropriate stage to intervene the underlying fibrotic pathways shared by various forms of ILD [38]. Recurrent infection and aspiration are likely the main factors, and in some cases, pleural parenchymal fibroelasticity is associated with recurrent infections [39]. The Safety and Efficacy of Nintedanib in Systemic Sclerosis (SENSCIS) trial [40] recently showed a therapeutic option for using antifibrotics in the management of SSc-ILD, which showed a lower annual decline of FVC in the nintedanib group compared to that in the placebo group, approved as a treatment for SSc by the FDA in 2019. A recent study chose an absolute decline in the FVC of ≥5% over 1 year and an absolute decline in DL_CO_ (corrected for Hb) of ≥10% within 1 year of follow-up as criteria for disease progression in progressive fibrosing ILD (PPF) [41]. The ATS/ERS guidelines recommend nintedanib for the treatment of progressive pulmonary fibrosis (PPF) in patients who have failed standard management for fibrotic ILD [41]. A recent double-blind, randomized, placebo-controlled trial assessed the safety and efficacy of pirfenidone in patients with SSc-ILD [42].

Immunosuppressive therapy with cyclophosphamide, MMF, or MTX has been an important treatment strategy for SSc and SSc-ILD [17]. These have been effective in preventing the progression of skin thickness and ILD; therefore, they have been proposed as the main treatments for SSc [43,44]. In the United States, 30.8% of patients with SSc receive immunomodulatory therapy during the first year after diagnosis [45]. In Korea, only a small number of patients were found to be treated with these drugs, presumably because they were not covered by national health insurance. Korean national insurance only covers azathioprine as an immunomodulatory therapy. MMF could be used in specific situations since 2020. Nintendanib is not covered yet. Therefore, not all patients were treated with these drugs according to published national guidelines. This was one of the limitations of our study.

Therefore, it is important to continuously monitor patients’ symptoms, conduct regular lung function tests regardless of the initial disease stage, and initiate medical treatments in the early phase.

This study has several limitations. The sample size was small, especially for cases of mortality; therefore, we could not compare mortality rates between the treatment groups and certain known risk factors such as autoantibodies did not show statistical significance. Second, patients who underwent PFT more than twice were included, which could have introduced a bias. Third, the study was retrospectively designed; therefore, certain subjective symptoms may not be exact. Moreover, recent novel biomarkers, such as IL-6 and KL-6, were not included in the analysis. Despite these limitations, we investigated the clinical outcomes and risk factors of SSc-ILD, which is a rare disease, in the long term.

## Conclusions

In summary, we found that the prevalence rates of common comorbidities, such as ILD, are compatible with other Asian studies. Even if the initial state of the patient was limited, approximately 10% of the patients showed progression. Furthermore, ILD is observed at a median of less than 5 years from the first visit and is known as a risk factor for mortality in patients with SSc; therefore, careful monitoring of the patients’ symptoms and signs is necessary. Further large cohort studies are needed to validate these risk factors and prevent disease progression in patients with SSc-ILD.

## Data Availability

The data sets analyzed for this study are not publicly available due to the data protection policy of the institution, however, are available upon special request.
